# PSP/reg and NT-proCNP to predict the occurrence of ICU-acquired sepsis in severe trauma patients: results of a pilot study

**DOI:** 10.1186/cc11783

**Published:** 2012-11-14

**Authors:** M Dupin, M Chanteperdrix, I Chaillol, A Pachot, F Venet, G Monneret, B Allaouchiche, A Gouel-Chéron

**Affiliations:** 1 bioMérieux, Lyon, France; 2 Hospices Civils de Lyon, France

## Background

Major trauma is characterized by a proinflammatory response, followed by an immunosuppression, increasing the risk for ICU-acquired infection. Several prognostic markers of sepsis in ICU patients have been identified and need to be assessed. Recently, Pancreatic Stone Protein/regenerating protein (PSP/reg) was shown to be increased during post-traumatic sepsis [1]. The N-terminal fragment of the C-type natriuretic peptide precursor (NT-proCNP) was assessed as a predictor of sepsis in multiple traumatized patients without brain injury [2]. The main objective of this study was to determine whether measurements of those biomarkers could help in the prediction of sepsis in severe trauma patients.

## Methods

This retrospective observational study was carried over 24 months in a trauma ICU at a university hospital. Trauma patients under mechanical ventilation with an Injury Severity Score (ISS) >25 and age >18 were included. Patients dying in the first 48 hours after trauma, having pulmonary inhalation/gut perforation during trauma or after the onset of infection, were excluded. We used already described ELISA protocols for the detection of PSP/reg [1] and NT-proCNP (Biomedica, BI-20872). Both biomarkers have been measured every 2 days from day 1 to day 4 after trauma. After descriptive analysis of clinical data (using medians with interquartile ranges), we evaluated the standard fold-change (SFC) and the area under the curve (AUC) between patients being infected (sepsis group) and patients not being (nonsepsis group).

## Results

We analyzed 61 trauma patients: age 37 (25; 50), ISS 38 (33; 45). Among them, 24 developed sepsis in the first week after trauma (pneumonia; median delay 4 days). There were no differences between sepsis and nonsepsis groups at admission regarding demographic data. Neither PSP/reg nor NT-proCNP showed significant differences between sepsis and nonsepsis groups, whatever time point was considered (respectively days 1 to 2 and days 3 to 4; Table 1). The computed values for SFC and AUC were lower than the minimal detectable values.

**Table 1:** PSP/reg and NT-proCNP doses (ng/ml) in function of patient groups

		Sepsis	Nonsepsis
PSP/reg	Days 1 to 2 (*n *= 38)	(*n *= 16) 30.1 (15.9; 51.4)	(*n *= 22) 27.8 (18.0; 41.3)
	Days 3 to 4 (*n *= 36)	(*n *= 7) 57.9 (21.7; 131.1)	(*n *= 29) 38.5 (15.8; 71.2)
NT-proCNP	Days 1 to 2 (*n *= 37)	(*n *= 16) 4.5 (3.6; 5.7)	(*n *= 21) 5 (3.8; 8.8)
	Days 3 to 4 (*n *= 36)	(*n *= 7) 3.6 (3.3; 3.9)	(*n *= 29) 4.7 (3.3; 5.6)

Data presented as median (Q1; Q3).

## Conclusion

In this pilot study, neither PSP/reg nor NT-proCNP can help in the prediction of sepsis in severe trauma patients. Contrary to the results published by Bahrami and colleagues [2], we did not show significant difference between sepsis and nonsepsis groups in patients without brain injury. Our study illustrates the complexity of validating biomarkers for sepsis prediction in independent cohort of patients.
